# Social pain and social gain in the adolescent brain: A common neural circuitry underlying both positive and negative social evaluation

**DOI:** 10.1038/srep42010

**Published:** 2017-02-07

**Authors:** Tim Dalgleish, Nicholas D. Walsh, Dean Mobbs, Susanne Schweizer, Anne-Laura van Harmelen, Barnaby Dunn, Valerie Dunn, Ian Goodyer, Jason Stretton

**Affiliations:** 1 Medical Research Council Cognition and Brain Sciences Unit, Cambridge, CB2 7EF, UK; 2 School of Psychology, Faculty of Social Sciences, University of East Anglia, Norwich, NR4 7TJ, UK; 3 Humanities and Social Sciences, California Institute of Technology, 1200 E. California Blvd., MC 228-77, Pasadena, CA 91125, USA; 4 Developmental Psychiatry Section, Department of Psychiatry, University of Cambridge, Cambridge, CB2 8AH, UK; 5 Washington Singer Laboratories, University of Exeter, Exeter, EX4 4QG, UK

## Abstract

Social interaction inherently involves the subjective evaluation of cues salient to social inclusion and exclusion. Testifying to the importance of such social cues, parts of the neural system dedicated to the detection of physical pain, the dorsal anterior cingulate cortex (dACC) and anterior insula (AI), have been shown to be equally sensitive to the detection of social pain experienced after social exclusion. However, recent work suggests that this dACC-AI matrix may index *any* socially pertinent information. We directly tested the hypothesis that the dACC-AI would respond to cues of *both* inclusion and exclusion, using a novel social feedback fMRI paradigm in a population-derived sample of adolescents. We show that the dACC and left AI are commonly activated by feedback cues of inclusion and exclusion. Our findings suggest that theoretical accounts of the dACC-AI network as a neural alarm system restricted within the social domain to the processing of signals of exclusion require significant revision.

Humans are fundamentally social. We create and reside within a diversity of emergent social systems ranging from couples, families, and groups to cities, countries and civilizations. These social structures have evolved in tandem with biological and psychological mechanisms that support social behavior, and the consequent rich capacity for social interaction has enabled humans to survive, reproduce, and flourish. Central among these mechanisms is the ability to detect and respond to diverse signals of social inclusion and social exclusion – behavioral dynamics that are critical to the establishment and maintenance of relationships, groups and social hierarchies. Indeed, acceptance by our desired social partners is so fundamental that social exclusion has profound negative consequences for affect, health and well-being^1^,^2^ and is particularly toxic during adolescence^3^.

There is burgeoning evidence to indicate that the mental ‘pain’ described by those experiencing such exclusion is more than just a metaphor. Brain imaging data suggest that the neural response to social rejection co-opts components of the well-established physical pain signature in the brain^4^. Cues of rejection have reliably been shown to activate a network of so-called ‘social pain’ regions that overlaps with the neural response to nociceptive stimulation and primarily includes the dorsal anterior-cingulate-cortex (dACC) and the anterior insula (AI)^5^,^6^,^7^,^8^,^9^. This account has been extended to suggest that the dACC in particular is involved in domain-general processing of pain information as it pertains to survival-relevant goal conflicts such as hunger or thirst and that social exclusion represents just one form of such survival threat^8^.

However, recent neuroimaging investigations within the social domain, have raised interesting questions about this social pain account of dACC-AI functioning^10^,^11^. For example, multi-level kernel density meta-analyses^10^ of the two prototypical social rejection paradigms used in neuroimaging studies – Cyberball (in which participants are excluded in a virtual ball-tossing game), and the reliving of memories of romantic rejection – have provided equivocal support for the claim that rejection activates the same neural matrix identified in studies of physical pain. Consistent with this, multivariate functional magnetic resonance imaging (fMRI) pattern analyses suggest that separate neural representations code physical pain and mental pain within this identified shared network^11^. Parallel to this, some have argued the broad dACC-AI overlap between social pain and physical pain can be simply explained as salience, and hence trigger multimodal cognitive processes involved in detecting, orienting attention towards, and/or reacting to salient events^12^.

An alternative possibility is that this pattern of dACC-AI co-activation emergent from the social exclusion literature is not simply a form of ‘pain’, but instead a more sophisticated index of the social dynamic^10^. One compelling candidate is that this network operates as a gauge of social inclusivity, a form of sociometer^13^,^14^. If true, then this system would subserve the processing of any signal that provides salient information about social inclusivity, whether it indexes social pain or ‘social gain’.

We therefore investigated the hypothesis that the dACC-AI matrix prototypically identified in studies of social rejection is in fact critically involved in processing signals of *both* social pain and social gain. We used a novel Social Feedback fMRI task that provides participants with comparably intense signals pertaining to either social exclusion or social inclusion, within the same paradigm, thus allowing us to identify their common and discrete neural substrates.

Participants believed that they were competing with other contestants in a multi-round game. Participants were told that: at the end of each round, one contestant is excluded from the game while the others are included in the next round; each round involves each contestant individually performing a social task and performance is evaluated by a panel of judges; that these ratings form the basis of the inclusion/exclusion decisions; and that the game is played in a hyperscanning context^15^ where each contestant is in a separate MRI scanner. In fact the judges and other contestants were confederates, only the participants were in a scanner, and only one round of the game, comprising our Social Feedback Task (Fig. 1; see also Supplemental Information), was ever played.

## Results

Our results showed that participants rated negative social feedback as more upsetting than neutral feedback (*t* = 12.6, df = 55, p < 0.001) and positive feedback as less upsetting than neutral (*t* = 13.5, df = 55, p < 0.001), as expected (Fig. 2A). Consistent with the social pain literature^5^,^6^,^7^, the fMRI data (all whole brain, *p* < 0.05, FWE corrected) revealed greater activation in the bilateral dACC and left AI when receiving negative compared to neutral social feedback (Fig. 2B). Critically, however, these same regions were also activated when receiving positive (relative to neutral) social feedback, along with the ventromedial prefrontal cortex (vmPFC) and ventral striatum bilaterally (Fig. 2C). In fact, there were no regions that were significantly more activated for negative social feedback relative to positive (negative > positive contrast), even when we explored the data using lower activation thresholds (*p* < 0.005, uncorrected). Furthermore, the reverse contrast (positive > negative) simply revealed activations in the aforementioned ventral striatum and vmPFC regions, areas traditionally associated with reward processing (Fig. 2D)^16^ (Table S2). These findings indicate that a common dACC-AI network subsumes the processing of information pertaining to both social exclusion and social inclusion.

This was supported by a logical ‘AND’ conjunction analysis^17^ of ‘negative > neutral social feedback (Fig. 2B)’ AND ‘positive > neutral social feedback (Fig. 2C)’, which revealed clusters in the left AI (peak voxel x = −28, y = 18, z = −10) and the dACC (peak voxel x = 2, y = 32, z = 24) that were significantly active across both conditions (whole brain *p* < 0.05, FWE corrected; Fig. 3A). The conjunction contrast was masked inclusively using the contrasts ‘positive feedback > baseline’ and ‘negative feedback > baseline’ (see Fig. S6) to ensure activation was not a product of the neutral condition, though it should be noted the results were the same without masking the conjunction. Furthermore, separate psychophysiological interaction (PPI) functional connectivity analyses, seeded from these AI and dACC regions, showed comparable results for the positive and negative social feedback conditions (relative to neutral feedback) with significant (p < 0.05, FWE corrected) associations with activity in the right fusiform gyrus and inferior occipital lobe for both contrasts (Supplemental Information, Table S3, and Fig. S1).

Is the common dACC-AI network identified here the same as that emerging from prior studies of social rejection^10^,^18^? An overlay of the results of our conjunction analysis (Fig. 3A) on the clusters identified in the whole brain meta-analysis of social rejection studies^10^ suggested that the conjunctive regions identified in the current data map closely onto the meta-analytic findings (Fig. 3B), indicating that our Social Feedback Task is activating the same network as the Cyberball and romantic rejection paradigms reviewed therein. A similar overlay (Fig. 3C), this time using just the results of our positive > neutral social feedback contrast, confirms that the network specifically underlying responses to positive evaluative information in the present data conjoins the social rejection network identified in the meta-analysis. In fact, if we extract the parameter estimates from our data that correspond to the peak dACC and AI coordinates from this whole-brain meta-analysis, they show the greatest activation during *positive*, rather than negative feedback in our data, suggesting that this network (hitherto associated with social pain) is actually more strongly activated in a social inclusion context. This is replicated when plotting the peak coordinates from a meta-analysis of social rejection tasks with a restricted focus on ACC activity^18^ (Supplemental Information, Fig. S2) and in structural and functional region of interest (ROI) analyses of the same regions (Supplemental Information).

Are there other potential accounts of the present data that merit consideration? One possibility (see Supplemental Information for a full discussion) is that the dACC-AI network activation found here in the context of signals of social inclusion simply occurs as a result of expectancy or carry-over effects from negative social feedback elsewhere in the task. However, these putative influences would also be present for the neutral feedback trials, for which the relevant activations were subtracted out in our critical positive social feedback contrast term, making this explanation less compelling. A related possibility is that positive and negative feedback activate a common neural network because they both involve some form of expectancy violation^19^,^20^. However, again this seems unlikely because, if for illustration we focus on the critical positive feedback findings, the pattern of dACC-AI activation remains even for the subset of participants (*n* = 10) who (by their own ratings) expected to be consistently judged as best across all social domains and for whom the positive feedback was therefore unlikely to violate expectancies (Fig. S3A).

**Figure 1:**
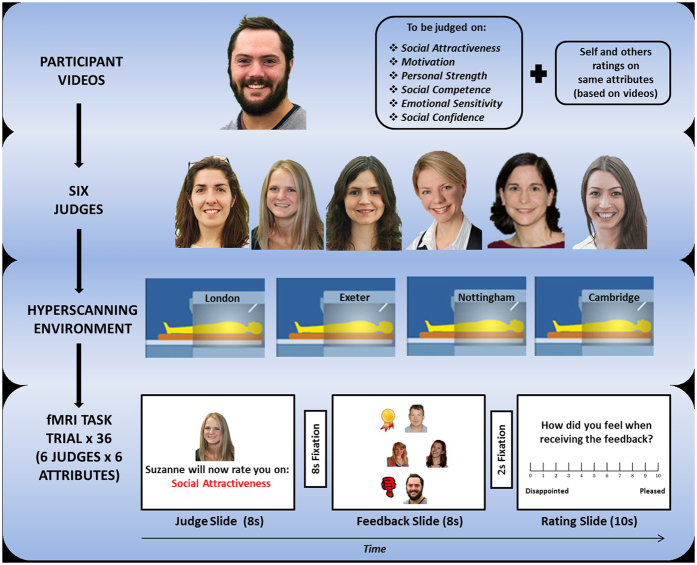
The Social Feedback Task. Participants (*n* = 56; Table S1) were first instructed to make 1-minute videos in which they talk freely about themselves and their aspirations. Participants believed their performance in these videos was being rated on six social attributes by a panel of six judges (confederates) in comparison to 3 other contestants (also confederates). Participants believed that they and the other contestants were in MRI scanners across the UK (in fact, only the participants were being scanned). They were able to view (and rate) the videos of the other contestants (confederates) prior to entering the scanner^13^. In the scanner, in order to provide signals of social inclusion and social exclusion, participants received feedback from each judge on each social attribute relative to the other contestants and were then asked to rate how the feedback made them feel. Feedback was either socially positive/inclusive (rated the best of the four contestants on that attribute by that judge and positioned at the top of the Feedback Slide with an adjacent gold medal), socially negative/excluding (rated the worst on that attribute and positioned at the bottom of the Slide with a thumbs-down graphic), or neutral (rated intermediate and positioned in the middle of the Slide). Participants received equal amounts of such positive, negative and neutral social feedback during the course of the task.

The shared dACC-AI activation also seems unlikely to be a simple function of emotional arousal as the effects remain after regressing out skin conductance responses (a reliable marker of psychophysiological arousal^21^ recorded during the feedback epochs (Fig. S4). Similarly, applying an exclusive mask of the neural correlates of rating the affective impact of feedback (Rating Slide; Fig. 1) still revealed significant dACC-AI feedback conjunction clusters, suggesting that this shared activation is not simply attributable to affect processing (Table S4). Analogously, the pattern of dACC-AI activation in our feedback conjunction remained after applying either an exclusive meta-analysis mask of ‘salience’ (from neurosynth.org)^19^,^22^ (Fig. S3B), or a mask created by re-binning the feedback trials as a function of trial-by-trial stasis/change in social rank^23^,^24^ (Fig. S5), suggesting that simple explanations based on general salience or social rank processing are also unlikely to account for the results.

## Discussion

The dACC and AI regions of the brain are implicated in a diverse range of psychological processes^17^. Within social contexts, the dACC-AI network has hitherto been associated with experiences of social exclusion and rejection^5^,^6^. However, our results show comparable patterns of involvement of this network in the processing of signals of social inclusion and of social acceptance. By comparable patterns, we mean we report significant independent contrasts of positive versus control conditions (either neutral or low-level baseline), and negative versus control conditions, and a significant conjunction (FWE corrected) for those separate effects. We are not intending to imply that those effects are identical in magnitude, although we failed to find any support for greater activation in this network in the face of negative social feedback relative to positive, or vice versa. These findings suggest that theoretical accounts of the dACC-AI network as a neural alarm system targeted at processing signals of exclusion within social contexts, and the resultant mental pain, require significant revision and extension. The current data are more consistent with a framework in which the dACC-AI matrix indexes signals of social inclusivity more generally within social contexts - a neural sociometer^13^. This accords with functional level models emphasizing the integration of signals of social inclusion and exclusion as a gauge of fluctuating social status^12^, and mirrors a similar theoretical shift concerning the brain’s so-called physical pain networks which have also been shown to be heavily implicated in the processing of physical pleasure^25^.

Several notable strengths of the current study bolster confidence in these conclusions, including the relatively large (for fMRI) and population-derived sample (*n* = 56), the use of a novel task targeted at the key research question, the application of a comprehensive analytic approach to address common potential confounds in the social pain literature^17^, and the stringent use of familywise error-corrected statistics.

Other findings from the wider social neuroscience literature are also consistent with this view that the prototypical dACC-AI social pain network is involved in the processing of inclusive social signals. Somerville *et al*.^20^, in a study ostensibly examining social feedback in the context of expectancy violations, report comparable levels of dACC activation when subjects viewed pictures of people who reportedly dislike them *or* like them^20^. Similarly, rostral ACC activation increases in tandem with increasing expectation of positive social feedback^26^. Furthermore, μ-opioid receptors (MOR) that moderate physical pain appear to respond to positive social feedback in key social pain structures^25^. Using Positron Emission Tomography (PET) combined with a social feedback task examining whether one is liked (social acceptance) or disliked (social rejection), MOR activation during social rejection was positively correlated with MOR activation during social acceptance in the anterior insula (left, r^2^ = 0.79; right, r^2^ = 0.62) and dACC (left, r^2^ = 0.86; right, r^2^ = 0.92) with no significant differences in levels of MOR activation in these structures between positive or negative social feedback conditions^27^.

Interestingly, potentially inconsistent data come from studies using the prototypical social rejection paradigm – Cyberball^4^,^5^,^6^,^28^. Cyberball invariably contains a social ‘inclusion’ comparison condition where the participant is included in the virtual ball tossing game. Unlike social rejection, though, inclusion within Cyberball does not appear to activate the dACC-AI network^5^,^6^,^7^. However, ‘inclusion’ here simply means not being excluded from the virtual ball tossing game. Such inclusion in this game playing context would be considered the social norm^20^ and consequently is unlikely to be overt or salient enough to markedly activate any putative inclusivity-related brain network. To address this, some studies have adapted Cyberball by using an ‘over-inclusion’ condition in which participants receive the ball 80% of the time^26^. However, if inclusion is the social norm as opposed to an overt, socially positive event, then increasing the number of social inclusion trials is likely to simply accentuate this. Indeed, although the fMRI results in these over-inclusion studies mirrored the usual Cyberball findings in showing that the dACC was more active during exclusion compared to over-inclusion, there was no behavioural difference in the level of subjective social pain reported between inclusion and over-inclusion conditions. This suggests that participants did not find over-inclusion any more socially rewarding than standard inclusion. Hence, while the Cyberball paradigm creates valid socially painful experiences through exclusion, it may be ill-suited to assess the social pleasure associated with inclusion.

**Figure 2:**
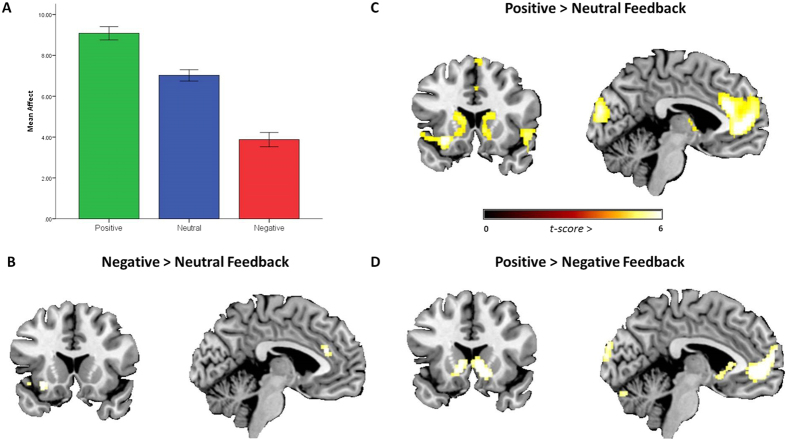
The Social Feedback Task results. (**A**) As expected, participants rated positive social feedback as more pleasing, and negative social feedback as more upsetting than neutral feedback (F(2,110) = 226.46, p < 0.001). (**B**) Negative (relative to neutral) social feedback revealed activation in the AI and dACC. (**C**) Positive (relative to neutral) social feedback revealed activation in the vmPFC, ventral striatum, AI and dACC. (**D**) Activation in the vmPFC and ventral striatum was observed when comparing positive relative to negative social feedback. No regions were significantly activated for negative > positive feedback. All results are whole brain t-values, p < 0.05, FWE corrected. Bars represent standard error of the mean.

**Figure 3:**
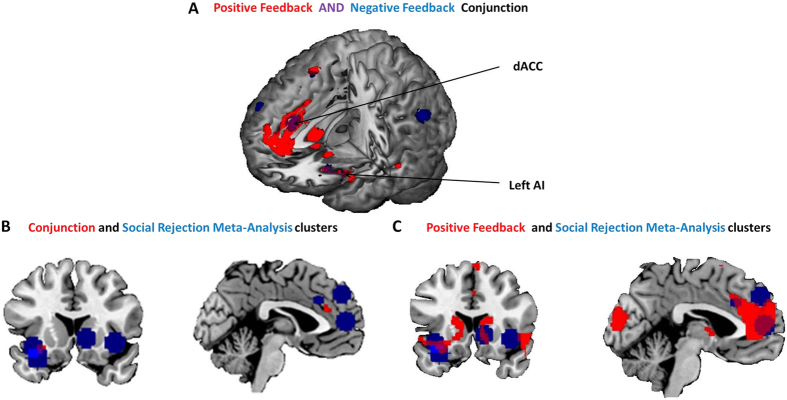
Conjunction analysis results. (**A**) A logical ‘AND’ conjunction of positive and negative social feedback (relative to neutral feedback) showed common activation of the AI and the dACC (t-values p < 0.05, FWE corrected). (**B**) Results of the conjunction analysis (red) overlaid onto clusters identified in the meta-analysis of the social rejection literature (Blue)^10^. (**C**) Results of just the positive > neutral social feedback contrast overlaid with the same meta-analysis clusters shows a high degree of overlap in the dACC, ventral striatum, left AI and vmPFC.

Some potential limitations merit comment. With the absence of a non-social comparison condition we were unable to evaluate the social specificity of our data, and hence rule out interpretations within the broader context of salience processing^12^, although our findings remain even when exclusively masking brain regions prototypically associated with salience processing. Secondly, our GSR data suggest that the neutral condition was not equidistant from the negative and positive conditions in terms of elicited arousal, with the neutral condition being more similar to the negative condition. However, our findings remain the same when comparing positive and negative feedback to our low-level baseline (i.e., without the neutral condition) and when regressing out GSRs in the analyses, suggesting that this does not account for the results. Importantly, the neutral condition was well titrated in terms of its emotional impact relative to the positive and negative conditions (Fig. 2). Finally, as more resources and importance appear to be bestowed upon social evaluation in adolescence^3^, our data may not be generalizable to the adult population. Future research into such questions would be beneficial.

In summary, we show that the classic social pain network in the human brain, centered on the dACC and AI, shows similar patterns of sensitivity to signals of social inclusion as it does to social rejection. These findings have strong theoretical implications for our understanding of the role of this neural network in social cognition and are consistent with a neural sociometer that gauges the implications of all pertinent social information with respect to the organism’s social inclusion status.

## Methods

### Participants

Participants were adolescents/young adults [*N* = 60; Mean (SD) age = 18 (0.7), range 17–20 years; 31 females] recruited from the population-representative ROOTS cohort (Total *N* = 1143)^29^. We selected adolescents/young adults as we felt the Social Feedback Task would resonate strongly with that demographic. Inclusion criteria were: normal or corrected-to-normal vision; and English speaking. Exclusion criteria were: any history of neurological trauma resulting in loss of consciousness; current psychotropic medication use; current neurological disorder; current Axis 1 psychiatric disorder according to the Diagnostic and Statistical Manual of Mental Disorders (DSM-IV; ref. 30); presence of metal in body; diagnosed specific learning disability; or IQ < 85 on the Wechsler Abbreviated Scale of Intelligence (WASI; ref. 31).

Participants recruited to the study showed no significant selection bias compared to the total ROOTS sample in terms of gender ratio or socioeconomic status as assessed using the ACORN (A Classification Of Residential Neighbourhoods) geodemographic measure^32^ (http://www.caci.co.uk).

One participant was removed from further analysis due to a failure of imaging acquisition. Additionally, in the post-scan questioning (see below) three subjects reported some disbelief concerning the veracity of the cover story and were removed from all subsequent analyses, leaving a total of 56 participants for analysis (See Table S1 for participant data).

The study was carried out in accordance with the Declaration of Helsinki and Good Clinical Practice guidelines and approved by the Cambridgeshire Research Ethics Committee. All participants provided written informed consent.

### The Social Feedback Task

The paradigm was styled as a ‘Big Brother’ game, where participants competed against other contestants (in fact these were confederates) to impress a set of six judges (also confederates) on a series of tasks in order to win through successive rounds of the game (they were told that one contestant per round was rejected from the game) and to eventually win the game. During the study participants were told they would be competing against three other contestants who were each located in MRI scanners located across the U.K, electronically linked so they can play the game interactively (hyperscanning) (see also Fig. 1).

Participants were told that there would be three rounds of the game in total, with one person being rejected on each round until there was one winner remaining. In fact, this was a cover story and only one round of the game was played with all participants being voted off at the end of round one before being fully debriefed following a series of post-scan questions. For this first (and only) round of the game, each participant made a one-minute video recording to be rated by the panel of six judges. Participants were told that these ratings decided which contestant would be rejected from the game and who would progress to the subsequent (fictitious) rounds. The participants were told the six judges were together in a room at a separate location where they could be e-mailed the video recordings and from where they could submit their ratings. The participants were shown pictures of the six judges who were all just a few years older than the participants and were told that the judges had been extensively trained in making social judgments from video recordings.

For the video recording, participants were asked to describe themselves, talk about what they enjoy doing, say what is important to them in life, outline their aims and achievements and say what was the most important thing that has happened to them was. Participants were given time before the video was recorded to think about these issues, and were shown a video made by a previous participant as an example. A still photograph of each participant was taken at this point for subsequent use in the fMRI session. Participants were informed that, for round one of the game, the judges would be rating these videos on a series of social dimensions (social competence, motivation, self-confidence, personal strength, social attractiveness and emotional sensitivity) that had been reliably linked to social success, prosperity and satisfaction across the life course, and that had been reliably shown to be easily rated on the basis of short video clips. They were told that, for each attribute, each judge would rank the participant and the other three contestants in terms of who was the best on that attribute (positive social feedback), who was the worst on that attribute (negative social feedback), and who was intermediate (neutral feedback) on that attribute. The decisions of each judge for each attribute (36 sets of feedback) were then shown to each participant during the fMRI session, prior to the final decision about who was rejected from the game on Round 1. Participants were told the design of the game was intended to build tension, akin to the voting on ‘Big Brother’ style game shows.

To encourage believability in the other contestants, having made their own video recording, participants were asked to rate their competitors’ videos along the same social dimensions as the judges were using. Participants were told that each of the other contestants would be doing the same with their (the participant’s) video in the other contestants’ separate locations.

Participants were told that different recordings for Rounds 2 and 3 would be completed following potential success on Round 1 (which never in fact happened, but was described in order to maintain believability).

In the MRI scanner (See Fig. 1), each judgment epoch began with an 8-second ‘Judge Slide’ showing which judge would be judging which attribute (e.g. *David will now be judging you on social attractiveness*). This was followed by an 8-second anticipation period of fixation, and an 8-second ‘Feedback Slide’, showing whether each contestant was judged to be the best (positive feedback), intermediate (neutral feedback) or worst (negative feedback) on that particular attribute by that particular judge. Following this ‘Feedback Slide’, and a 2-second fixation, a 10-second ‘Rating Slide’ of how the participants felt about the feedback (ranging from 0 (disappointed) – 10 (pleased)) was completed. This sequence was repeated 36 times for each social attribute from each judge, resulting in 12 ‘best’ judgments, 12 ‘neutral/intermediate’ judgments and 12 ‘worst’ judgments. Attribute and judge orders were counterbalanced across participants. At the end of the 36 judgments, overall judgments were made by each judge detailing whether the participant had made it through to the next round, a total of 6 such final judgments was made (one by each judge); 5 of which were ‘worst’ and one ‘middle’ resulting in the participant being rejected on round one of the game. Following the scan, as a manipulation check, participants were asked a series of questions aimed at assessing believability of the task and of the hyperscanning environment.

All personally identifiable information (videos and photographs) was deleted immediately following debriefing.

### Data Acquisition and Analysis Approaches

#### Image acquisition and preprocessing

MRI scanning was conducted at the Medical Research Council Cognition and Brain Sciences Unit on a 3-Tesla Tim Trio Magnetic Resonance Imaging scanner (Siemens, Germany) by using a head coil gradient set. Whole-brain data were acquired with echoplanar T2*-weighted imaging (EPI), sensitive to BOLD signal contrast (48 sagittal slices, 3 mm thickness; TR = 2000 ms; TE = 30 ms; flip angle = 78°; FOV 192 mm; voxel size: 3 × 3 × 3 mm). To provide for equilibration effects the first 5 volumes were discarded. T1 weighted structural images were acquired at a resolution of 1 × 1 × 1 mm.

SPM8 software (www.fil.ion.ucl.ac.uk/spm/) was used for data analysis. The EPI images were sinc interpolated in time for correction of slice timing differences and realignment to the first scan by rigid body transformations to correct for head movements. Field maps were estimated from the phase difference between the images acquired at the short and long TE and unwrapped, employing the FieldMap toolbox. Field map and EPI imaging parameters were used to establish voxel displacements in the EPI image. Application of the inverse displacement to the EPI images served the correction of distortions. Utilising linear and non-linear transformations, and smoothing with a Gaussian kernel of full-width-half-maximum (FWHM) 8-mm, EPI and structural images were co-registered and normalised to the T1 standard template in Montreal Neurological Institute (MNI) space. Global changes were removed by proportional scaling and high-pass temporal filtering with a cut-off of 128 s was used to remove low-frequency drifts in signal.

#### Statistical analysis approach to fMRI data

After preprocessing, statistical analysis was performed using the general linear model. Analysis was carried out to establish each participant’s voxel-wise activation during the Feedback and Rating Slides (see Fig. 1). Activated voxels in each experimental context were identified using an epoch-related statistical model representing each of the three feedback trial types and subsequent affect ratings, convolved with a canonical haemodynamic response function and mean-corrected. Six head-motion parameters defined by the realignment were added to the model as regressors of no interest. Multiple linear regression modelling was then applied to generate parameter estimates for each regressor at every voxel. At the first level, the following feedback contrasts were generated; ‘positive feedback’; ‘neutral feedback’; ‘negative feedback’; ‘positive feedback’ minus ‘neutral feedback’; ‘negative feedback’ minus ‘neutral feedback’; ‘positive feedback’ minus ‘negative feedback’ and ‘negative feedback’ minus ‘positive feedback’. The same contrasts were also generated for the ratings of affect (Rating Slides) following each Feedback Slide. For group statistics, random effects analysis was utilized. A conservative voxel-wise statistical threshold of P < 0.05 familywise error (FWE) corrected for multiple comparisons across the whole-brain was used for all analyses.

## Additional Information

**How to cite this article:** Dalgleish, T. *et al*. Social pain and social gain in the adolescent brain: A common neural circuitry underlying both positive and negative social evaluation. *Sci. Rep.* **7**, 42010; doi: 10.1038/srep42010 (2017).

**Publisher's note:** Springer Nature remains neutral with regard to jurisdictional claims in published maps and institutional affiliations.

## Supplementary Material

Supplementary Information
