# Supplementary material for: Social pain and social gain in the adolescent brain: A common neural circuitry underlying both positive and negative social evaluation
Source: Sci Rep. 2017 Feb 7;7:42010. doi: 10.1038/srep42010 (PMC5294419; doi:10.1038/srep42010)
Supplement: Supplementary Information [file srep42010-s1.pdf]

# Supplemental Information

## Title: Social pain and social gain in the adolescent brain: A common neural circuitry underlying both positive and negative social evaluation

**Authors:** Tim Dalgleish[1†], Nicholas D. Walsh[2†], Dean Mobbs[3], Susanne Schweizer[1], Anne-Laura van Harmelen[4], Barnaby Dunn[5], Valerie Dunn[4], Ian Goodyer[4], Jason Stretton[1].

**Affiliations:**

[1] Medical Research Council Cognition and Brain Sciences Unit, Cambridge, CB2 7EF, UK.

[2] School of Psychology, Faculty of Social Sciences, University of East Anglia, Norwich, NR4 7TJ, UK.

[3] Humanities and Social Sciences, California Institute of Technology, 1200 E. California Blvd., MC 228-77, Pasadena, CA 91125, USA.

[4] Developmental Psychiatry Section, Department of Psychiatry, University of Cambridge, Cambridge, CB2 8AH, UK.

[5] Washing Singer Laboratories, University of Exeter, Exeter, EX4 4QG, UK.

[†] These authors should be considered as joint first author

**Corresponding Author**: Tim Dalgleish, Medical Research Council Cognition and Brain Sciences Unit, Cambridge, CB2 7EF, UK. Tel: 01223 273685 tim.dalgleish@mrc-cbu.cam.ac.uk

## Supplemental Materials and Methods

### Psycho-Physiological Interactions (PPI) analytic approach

A conjunction analysis revealed 2 clusters that were comparably significantly active during valenced social (positive and negative) feedback compared to neutral feedback; the left anterior insula (AI) (peak voxel; x=-28, y=18, z=-10) and the dorsal anterior cingulate cortex (dACC) (peak voxel; x=2, y=32, z=24) (Fig. 3A, main paper). In order to determine whether there was differential functional connectivity between feedback conditions, we created two 10-mm sphere volumes of interest (VOI) centred at the peak voxel for each of the above specified regions. Next, the time-series for each participant, in each VOI, was computed by using the first eigenvariate from all voxels' time series. The BOLD time series for each participant was deconvolved to estimate a 'neuronal time series' for this region.

The psycho-physiological interaction term (PPI regressor) was calculated as the element-by-element product of the VOI neuronal time series and a vector coding for: [1 = positive feedback vs -1 = neutral feedback] and [1 = negative feedback vs -1 = neutral feedback]. This product was reconvolved by the canonical hemodynamic response function (hrf). The model also included the main effects of task convolved by the hrf, and the movement regressors as effects of no interest. Participant specific PPI models were run, and contrast images generated for positive PPIs were conservatively thresholded at $p<0.05$ FWE corrected. The identified regions have greater connectivity with the source region according the context of the contrasts described above.

### Galvanic skin conductance (GSR) data acquisition

A Biopac MP 150 System together with Acqknowledge software (Biopac Inc., Goleta, CA) was used to record skin conductance data during task performance within the MRI environment. Two Ag-AgCl electrodes spread with electrolyte paste were positioned on the palm of the left hand. These were connected to a Biopac GSR100C module with the gain set to 5 microSiemens/V, the low pass filter to 1.0 Hz, and the high pass filters to DC. An MRI compatible version of the equipment was used (http://www.biopac.com/Manuals/mecmri.pdf). A continuous skin conductance signal was output into Acqknowledge 3.9 software on an analysis computer and time-stamped to indicate the onset and offset of each event by means of digital markers sent from the stimulus delivery computer. Data were acquired at 200 samples per second. The data were transformed into microsiemens (µS) prior to analysis.

**Galvanic skin response (GSR) data analytic approach**

Owing to technical difficulties with positioning of the electrode and loss of signal, 14 participants GSR were irretrievable, leaving 42 participants with usable GSR data. Due to non-normality of the data, a natural log (x+1) transform was applied to the raw data for each participant. The GSR to the Feedback Slide presentation was assessed as the base-to-peak difference with baseline being estimated using the mean signal across the 2 second period immediately prior to the Feedback Slide onset and the peak response being extracted from the period between Feedback Slide onset and offset [1,2]. Resultant GSR data are negative in value (see Supplemental Results) due to higher GSR during the anticipatory baseline (constant across feedback conditions) relative to feedback itself.

## Supplemental Results

### Psycho-physiological interactions (PPI)

We wanted to examine whether the functional connectivity of the identified shared dACC-AI network for positive and negative social feedback in the Social Feedback Task differed as a function of feedback type. A PPI analysis was therefore conducted to assess differential connectivity across feedback conditions ('positive' minus 'neutral'; 'negative' minus 'neutral') seeding from the each cluster identified in the conjunction presented in Fig. 3A (left AI and dACC). PPI maps were entered into an ANOVA with region (2 levels; left AI and dACC) and condition (positive and negative feedback) as independent variables. In each condition, both regions showed connectivity to the right fusiform gyrus (Fig. S1), likely reflecting the recognition of multiple faces [3] that were present in each of the Feedback Slides. There were no main effects or interactions of either region or condition. (See Table S3 for a full list of regions identified in the PPI analysis).

### Parameter estimates from each feedback condition in the Social Feedback Task derived from the social rejection meta-analytic dACC and AI coordinates (8, 18).

We wanted to confirm that the regions of the dACC-AI most strongly associated with social rejection within the meta-analytic literature showed at least comparable levels of activation in the positive feedback condition in our own data, relative to our negative feedback condition, as would be expected if there was a common neural substrate for signals of social rejection and inclusion.

We extracted the parameter estimates for each feedback condition in the Social Feedback Task (positive, neutral, negative) at the peak dACC (x=-4, y = 24, z= 32) and left AI (x=-36, y = 20, z= -10) coordinates identified in the Cacioppo et al. (2013) meta-analysis of social rejection tasks [4].

A repeated measures ANOVA comparing the magnitude of the parameter estimates for the dACC coordinates across the three feedback conditions in the Social Feedback Task revealed a significant main effect ($F(2,110)=10.46$, $p<0.005$). Post hoc tests (Bonferroni) showed significantly ($p<0.005$) greater dACC activation during both positive (mean = .16) and negative (mean = .0004) feedback compared to neutral feedback (mean = -.21). Critically, there was no significant difference between positive and negative feedback conditions (p=.13), with the parameter estimate in fact being numerically *higher* for the positive feedback condition. A similar pattern emerged for the left AI coordinate, with a main effect of condition ($F(2,110)=7.71$, $p<0.001$) driven by significantly (Post-hoc Bonferroni, $p<0.001$) greater left AI activation during both positive (mean = .19) and negative (mean =.16) feedback compared to neutral feedback (mean = -.07) (Fig. S2). Again, there was no significant difference between positive and negative feedback conditions (p=1) (Fig. S2).

We applied the same process to a more recent meta-analysis focusing on the contribution of the entire ACC to social pain [5]. The parameter estimates for each feedback condition in the Social Feedback Task were extracted for the peak coordinate of the ACC (converted to MNI space) (x=5, y = 40, z= -5) for social pain and social rejection across published Cyberball studies (Fig. S2). Here, repeated measures ANOVA again revealed a significant

main effect of feedback condition ($F(2,110)=10.07$, $p<0.005$). Post hoc tests (Bonferroni) this time showed significantly greater activation during positive (mean = .31) compared to negative (mean = .061) and neutral feedback (mean = .05). There was again no significant difference between neutral and negative feedback conditions ($p=1$).

**Region of Interest (ROI) Analyses for positive versus negative feedback conditions**

We additionally performed ROI analyses (using MarsBAR) of the dACC and AI using structural and functional masks. Structural ROIs were defined using the Harvard-Oxford atlas on the WFU Pickatlas. The dACC was limited in the y plane from y=-36 to y = 0. The AI was defined using the insula label combined with the 8mm spheres centered on the peak meta-analysis voxels described above for the left AI. We compared the dACC and AI activation directly between positive and negative feedback conditions for both structural and functional ROIs. There were no significant activations for the negative>positive contrast. The structural ROI of the dACC was significantly more active in the positive condition over the negative condition ($t=1.93$, $p=0.028$). Similarly, both the left ($p=0.059$) and right AI ($p=0.071$) were at trend for more activity during positive feedback than negative. The functional ROIs showed similar results, the dACC[4] ($t=1.82$, $p=0.03$) and ACC[5] ($t=2.57$, $p<0.005$) meta-analysis peaks were both significantly more active during positive feedback compared to negative feedback. The left AI[4] showed a trend towards greater activity in the positive relative to the negative condition ($p=0.08$).

**Supplemental analyses addressing potential alternative accounts of the current data**

A number of plausible alternative accounts of the involvement of the dACC-AI network in social pain have been promoted within the literature (see [6] for an excellent discussion). It is therefore important to address such explanations within the present data, in particular for the novel result showing activation of this network in the context of positive feedback.

**Trial and task context effects**

A putative explanation for the activation of the social pain network during the positive feedback condition in the Social Feedback Task is that it results from the presence of social rejection information on either the feedback trials themselves (participants always see another contestant being rejected) or within the overall task context (participants would expect rejection on future trials/will have experienced rejection on past trials). However, these potential effects are also all present for trials in which participants receive neutral feedback (there is always another contestant being rejected and the overall task context is the same). For this reason, all analyses of the positive feedback condition reported in the results subtracted out activation associated with neutral feedback to take account of these potential confounds. It therefore seems implausible that contextual rejection-related information can account for the dACC-AI activation to positive (relative to neutral) feedback.

Another related possibility is that positive social feedback on the task was in fact experienced as socially negative for some reason. Again this seems unlikely because participants rated positive feedback significantly more positively than negative or neutral feedback (Fig. 2A), and positive feedback activated traditional reward-related brain areas

(vmPFC and ventral striatum; [7] relative to both neutral (Fig. 2C) and negative (Fig. 2D) feedback, but this was not the case for the negative feedback condition.

**Expectancy violation**

A wealth of research has implicated the dACC in monitoring outcomes in relation to expectancies [8]. A possible account of the present data pertaining to positive social feedback on the Social Feedback Task (and indeed to negative feedback and to the broader findings of the social pain literature; (see [6] for a rejoinder to these arguments), is therefore that the patterns of dACC activation reported are a result of a greater violation of expectations, relative to the neutral contrast condition.

In order to address this possibility of expectancy violation, we ran a separate GLM consisting of the feedback contrasts (positive, negative and neutral) of only those participants ($n$=10) who, when ranking the videos of themselves and the other contestants at the beginning of the task (see Main Text; Materials and Methods), consistently rated themselves as the best across the entire set of social dimensions being judged. Our reasoning was that, for this subset of participants, being ranked first during the task itself (the positive feedback condition) would not represent a violation of expectancies as it simply confirmed their own views regarding the rank ordering of the participants. Consequently, if the conjunctive dACC region presented in Fig. 3A was associated with expectancy violation, it could be assumed that this region would be more active during negative feedback and less active during positive feedback (relative to neutral feedback) for these particular participants. However, the results indicated the reverse pattern - the dACC region remained significantly active during positive feedback (relative to neutral

feedback) in this subsample (using a lower threshold of $p<0.001$ uncorrected due to the restricted sample size) (Fig. S3) but not during negative feedback (even at a very low threshold of $p<0.05$ uncorrected). This suggests that expectancy violation does not adequately explain the activation of this region during social evaluation.

**Psychophysiological arousal as a potential confounding factor**

A potential account of the shared neural activation for positive and negative feedback, relative to neutral, on The Social Feedback Task is that these forms of valenced feedback are more emotionally arousing than neutral feedback. A similar criticism has been levelled at findings within the social pain literature (see [6] for a review and discussion). To examine this possibility we acquired event-related galvanic skin response (GSR) data in parallel with the fMRI data (see Supplemental Materials and Methods above for details of acquisition and analytic approach) as an index of autonomic arousal [1].

We first examined whether the profile of arousal across conditions was consistent with an arousal-based account of the dACC-AI co-activation for the positive and negative (relative to neutral) feedback events (Fig. S4). Inspection of the GSR data revealed a different profile (positive>neutral=negative) compared with the dACC-AI activation profile (positive>neutral<negative). This was confirmed using a repeated measures ANOVA which revealed a significant main effect of condition in the GSR data ($F(2,82)=7.63$, $p<0.05$). Follow-up paired sample t-tests revealed positive feedback elicited significantly greater GSR response than both neutral ($p<0.05$) and negative feedback ($p<0.05$) conditions (Fig. S4). The different profiles of activation for GSR and for dACC-AI activation across conditions suggest that the pattern of neural activation is not a simple

function of arousal as indexed by GSR. To further support this conclusion, we included the GSR data as a covariate of no interest in the fMRI data analyses, finding that it did not impact the results of the feedback conjunction analysis.

**Posterior probabilities of peak-coordinates** Neurosynth (http://neurosynth.org), a platform for large-scale, automated synthesis of functional magnetic resonance imaging (fMRI) data,allows the estimation of the posterior probability for the reverse inference of a given coordinate, representing the likelihood that activation in that region is involved in a given cognitive process. Strikingly, the highest posterior probability loadings for the dACC peak coordinates (x=2, y=32, z=24) identified in our conjunction analysis were for the terms 'abuse' (0.87), 'reward anticipation' (0.82), 'distraction' (0.81), 'monetary' (0.80), and 'nociceptive' (0.80), further suggesting that this region is implicated in processing information from both positive and negative domains. Similarly, for the left AI coordinate (x=-28, y=18, z=-10) the terms 'monetary reward' (.086), 'intense' (0.84) and consistency (0.83) were the highest posterior probability loadings, again reflecting the involvement of these regions in the evaluation of positive information.

**Tracking of social rank**

Our Social Feedback Task involves dynamic shifts in the ranking of the four contestants as a function of the successive positions allocated to them across the 36 judge/attribute feedback trials (Fig. 1). It is helpful therefore to verify that the dACC-AI conjunction that we find (Fig. 3A) is not simply a function of the tracking of such dynamic changes in rank [15,16]. Thus, we re-binned the data and ran a separate GLM on the neural response to the Feedback Slide with three conditions based on whether the participant has moved up,

down or stayed at the same ranking position (compared to the previous trial). A conjunction of moving up, moving down and staying the same rank ($p<0.05$ FWE corrected) was consistent with prior studies on social rank processing [15,16], showing medial prefrontal cortex, rostral ACC, precuneus and amygdala activation (Fig. S5). We saved this rank conjunction as an exclusive mask, again conservatively thresholded at $p<0.001$ uncorrected, and applied it to our positive and negative social feedback conjunction analysis. Importantly, both the dACC and AI clusters survived masking at $p<0.05$, FWE corrected.

References


1. Milad MR, Orr SP, Pitman RK, & Rauch SL Context modulation of memory for fear extinction in humans. *Psychophysiology* 42, 456. (2005)
2. Orr SP, & Roth TW Psychophysiological assessment: clinical applications for PTSD. *Journal of affective disorders* 61, 225. (2000)
3. Sergent J, Ohta S, & Macdonald B Functional Neuroanatomy of Face and Object Processing - a Positron Emission Tomography Study. *Brain* 115, 15.
4. Cacioppo, S. *et al.* A Quantitative Meta-Analysis of Functional Imaging Studies of Social Rejection. *Sci Rep-Uk* **3**, doi:Artn 2027 (1992)
5. Rotge, J. Y. *et al.* A meta-analysis of the anterior cingulate contribution to social pain. *Soc Cogn Affect Neurosci* **10**, 19-27, doi:10.1093/scan/nsu110 (2015).
6. Eisenberger, N. I. Social pain and the brain: controversies, questions, and where to go from here. *Annu Rev Psychol* **66**, 601-629, doi:10.1146/annurev-psych-010213-115146 (2015).

7. Fliessbach K, *et al.*, Social comparison affects reward-related brain activity in the human ventral striatum. *Science* 318, 1305. (2007)

8. Botvinick MM, Cohen JD, & Carter CS Conflict monitoring and anterior cingulate cortex: an update. *Trends Cogn Sci* 8, 539. (2004)

9. Etkin A, Egner T, & Kalisch R Emotional processing in anterior cingulate and medial prefrontal cortex. *Trends Cogn Sci* 15, 85. (2011)

10. Mechias ML, Etkin A, & Kalisch R A meta-analysis of instructed fear studies: Implications for conscious appraisal of threat. *Neuroimage* 49, 1760. (2010)

11. Shackman AJ, *et al.*, The integration of negative affect, pain and cognitive control in the cingulate cortex. *Nat Rev Neurosci* 12, 154. (2011)

12. Nichols, T., Brett, M., Andersson, J., Wager, T. & Poline, J. B. Valid conjunction inference with the minimum statistic. *Neuroimage* **25**, 653-660, doi:10.1016/j.neuroimage.2004.12.005 (2005).

13. Iannetti GD, Salomons TV, Moayedi M, Mouraux A, & Davis KD Beyond metaphor: contrasting mechanisms of social and physical pain. *Trends Cogn Sci* 17, 371. (2013)

14. Yarkoni, T., Poldrack, R. A., Nichols, T. E., Van Essen, D. C. & Wager, T. D. Large-scale automated synthesis of human functional neuroimaging data. *Nat Methods* **8**, 665-U695, doi:Doi 10.1038/Nmeth.1635 (2011).

15. Zink, C. F. et al. Know your place: neural processing of social hierarchy in humans. *Neuron* 58, 273-283, doi:10.1016/j.neuron.2008.01.025 (2008).

16. Kumaran, D., Melo, H. L. & Duzel, E. The emergence and representation of knowledge about social and nonsocial hierarchies. *Neuron* 76, 653-666, doi:10.1016/j.neuron.2012.09.035 (2012).

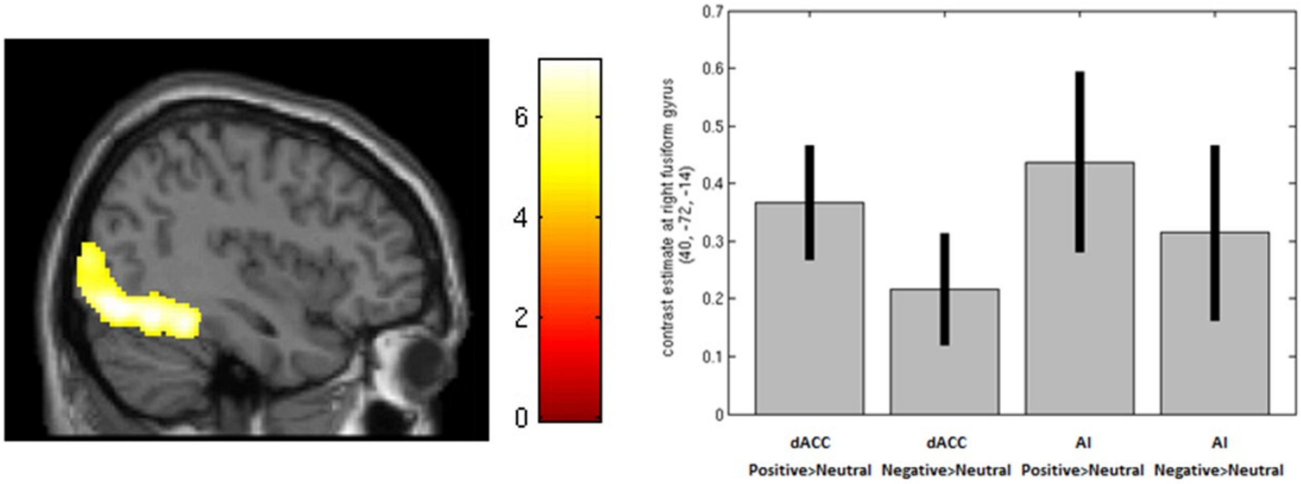


**Fig. S1.** PPI analysis. Significant co-activation (t-values, $p<0.05$ FWE corrected) of the right fusiform gyrus from each seed region (dACC and AI) and each condition (positive and negative feedback). The graph depicts the parameter estimates of the right fusiform gyrus for each seed region and condition. Bars represent $\pm$ 1 standard error of the mean.

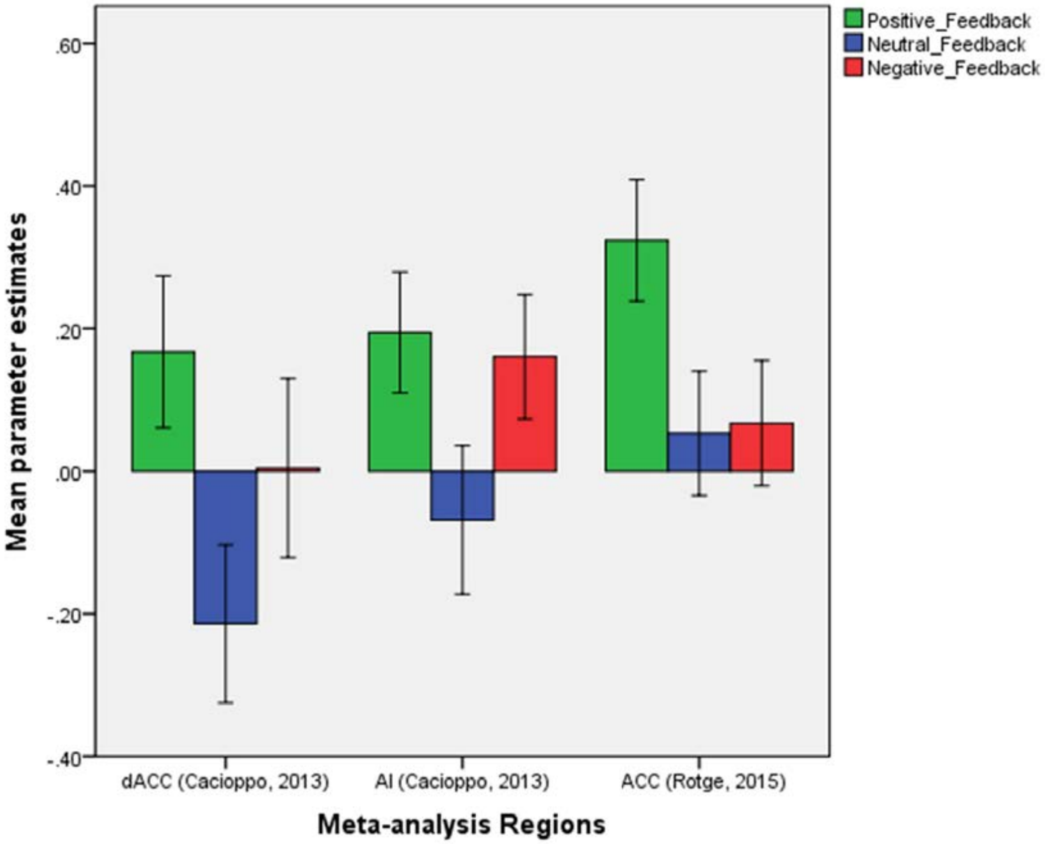


**Fig. S2.** Parameter estimates derived from social pain meta-analyses. Using the peak dACC and AI coordinates identified in the Cacioppo et al. (2013) meta-analysis ([4]), parameter estimates were extracted for each feedback condition in the Social Feedback Task. The estimates of the dACC and AI were significantly greater in the positive and negative feedback conditions compared to neutral feedback and critically did not differ significantly from each other. Using the peak ACC coordinate identified in the Rotge et al. (2015) meta-analysis ([5]), parameter estimates were extracted for each feedback condition. The estimates of the ACC were significantly greater in the positive feedback condition compared to negative and neutral feedback. Bars represent ± 1 standard error of the mean.

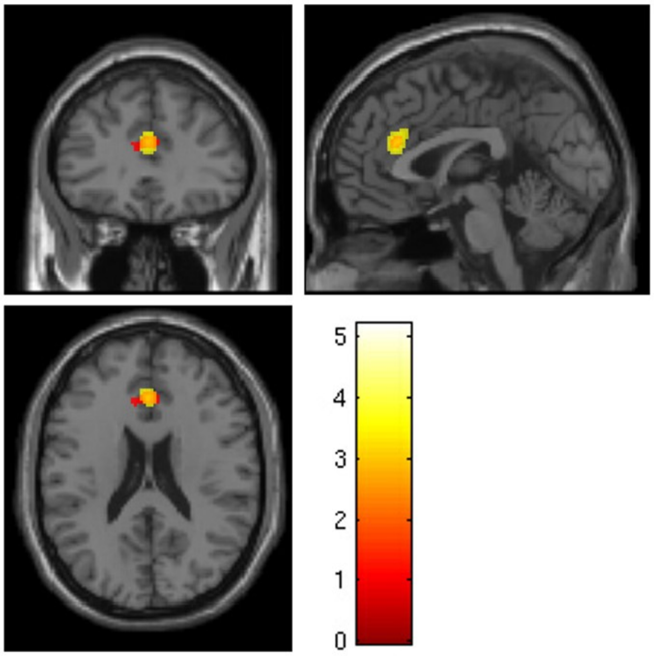


**Fig. S3.** Expectancy violation map. Activation of the dACC (t-values p<.0.001 uncorrected; shown in yellow) was observed during positive feedback (relative to neutral feedback) in a subsample (*n*=10) of participants who ranked themselves top (relative to the other contestants) across all social domains. This region conjoins (orange) with the main feedback conjunction analysis dACC region (red).

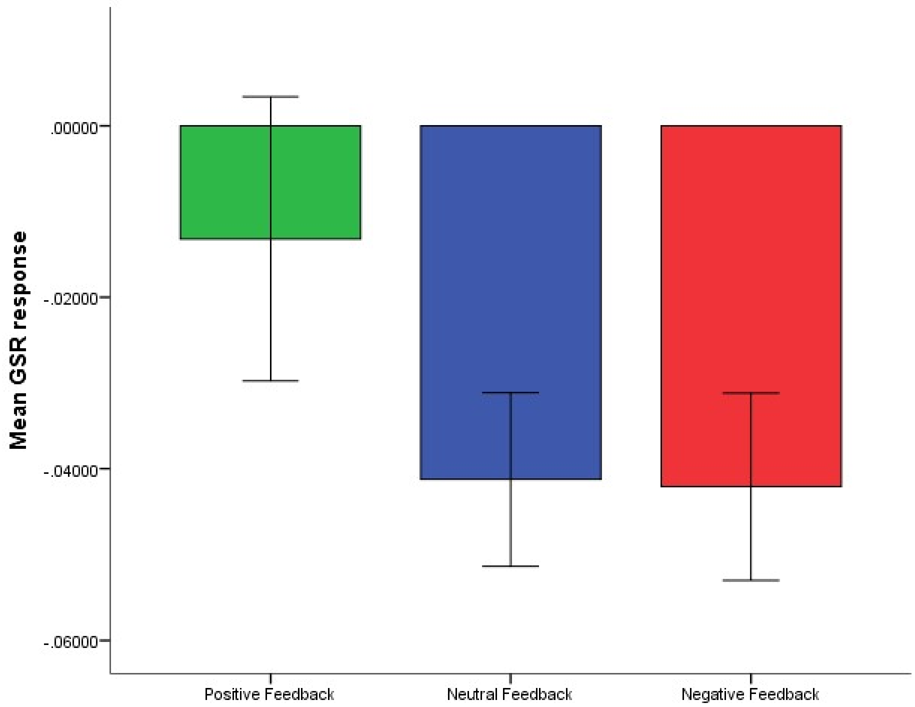


**Fig. S4.** Mean galvanic skin response (GSR) in microsiemens (relative to the anticipatory baseline) during each feedback condition. Bars represent ± 1 standard error of the mean.

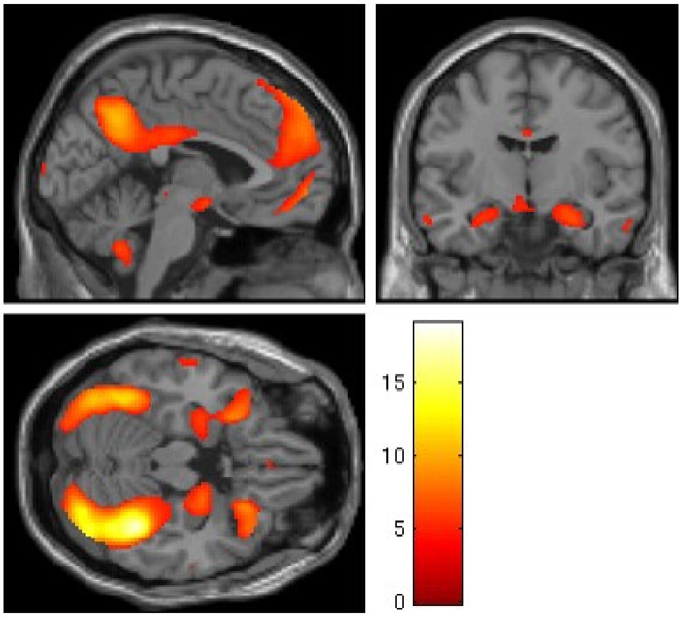


**Fig. S5.** Social rank analysis. A conjunction of moving up rank, moving down rank and maintenance of rank on the Social Feedback Task revealed activation bilaterally in the orbitofrontal cortex, amygdala, hippocampus, fusiform gyrus and in the dorsomedial prefrontal cortex extending into the rostral ACC and precuneus (t-values $p<0.05$, FWE corrected).

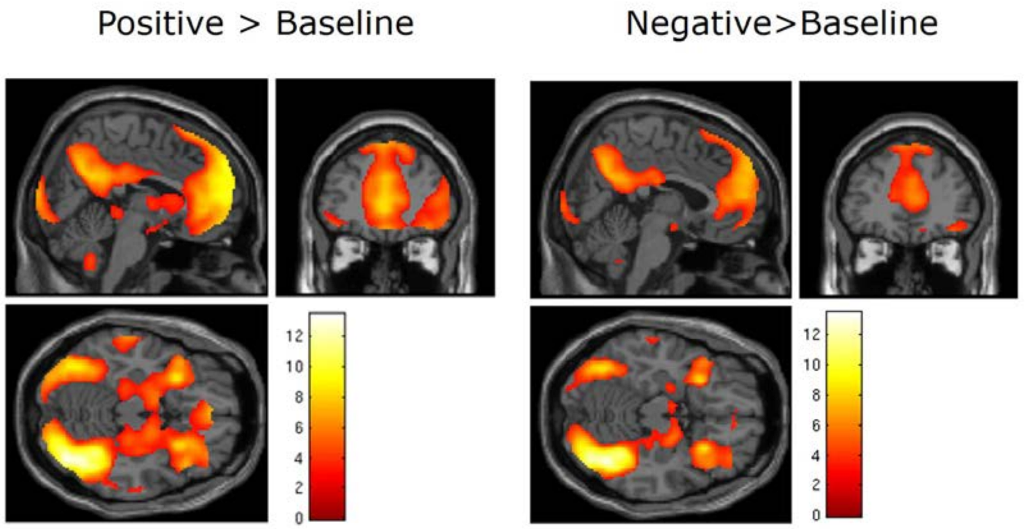


**Fig. S6.** Feedback versus Baseline. To ensure specificity of activation from both positive and negative feedback conditions, the contrasts of ‘Positive Feedback > Implicit Baseline’ and ‘Negative Feedback > Implicit Baseline’ were used as inclusive masks (t-values $p<0.001$ uncorrected, MNI coordinates shown at; $x = 4$, $y = 42$, $z = -16$) on the conjunction analysis shown in Figure 3 (main manuscript). Activation of the bilateral insula, medial frontal gyrus, anterior cingulate and visual areas are common to both contrasts, ensuring the result of the conjunction were not simply a product of the neutral condition. n.b. there were no surviving voxels for the contrast “‘negative > baseline’ > ‘positive > baseline’” even when we explored the data at a reduced threshold of $p<0.05$ uncorrected.

**Table S1.** Characteristics of the sample.

| | Sample |
|---|---|
| N = | 56 |
| Age [Years (SD)] | 17.7 (2.3) |
| Gender (M/F) | 25/31 |
| Socioeconomic status (ACORN) [N/%] | |
| Wealthy/urban | 32 (57%) |
| Comfortable | 14 (25%) |
| Moderate means/hard-pressed | 9 (18%) |
| WASI IQ [Mean (SD)] | 107 (9.4) |

WASI= Wechsler Abbreviated Scale of Intelligence; ACORN = A Classification Of Residential Neighbourhoods.

**Table S2.** Brain regions activated during social feedback.

| Contrast | Region | MNI Coordinates (X,Y,Z) | *k*-voxel | *z*-score | *p*-value* |
|---|---|---|---|---|---|
| Positive > Neutral | | | | | |
| | L Anterior Cingulate | 0, 32, 24 | 1218 | 6.57 | <0.05 |
| | R Cerebellum | 26, -80, -38 | 285 | 6.14 | <0.05 |
| | L Insula | -28, 16, -12 | 85 | 5.85 | <0.05 |
| | R Caudate | 8, 10, -2 | 109 | 5.63 | <0.05 |
| | L Cuneus | -4, -92, 18 | 145 | 5.40 | <0.05 |
| | L Middle Frontal Gyrus | -28, 48, 28 | 297 | 5.40 | <0.05 |
| | L Cerebellum | -26, -80, -34 | 36 | 5.15 | <0.05 |
| | L Caudate | -12, 14, 10 | 24 | 4.88 | <0.05 |
| | R Insula | 48, 14, -4 | 46 | 4.83 | <0.05 |
| Negative > Neutral | | | | | |
| | L Insula | -28, 16, -12 | 9 | 4.97 | <0.05 |
| | L Anterior Cingulate | 2, 32, 24 | 19 | 4.81 | <0.05 |
| | L Inferior Parietal Lobe | -60, -52, 38 | 5 | 4.67 | <0.05 |

| | | | | | |
|---|---|---|---|---|---|
| Positive > Negative | | | | | |
| | R Caudate | 8, 12, -6 | 330 | 6.00 | <0.05 |
| | L Putamen | -10, 8, -8 | 330 | 5.96 | <0.05 |
| | L Anterior Cingulate | -10, 48, 0 | 502 | 5.61 | <0.05 |
| | L Cerebellum | -36, -80, -24 | 184 | 5.58 | <0.05 |
| | R Middle Occipital Lobe | 32, -82, 8 | 111 | 5.40 | <0.05 |
| | R Cuneus | 10, -94, 30 | 38 | 5.09 | <0.05 |
| | L Cuneus | -2, -100, 12 | 23 | 4.95 | <0.05 |
| | L Middle Occipital Lobe | -30, -92, 10 | 19 | 4.95 | <0.05 |
| | L Superior Frontal Gyrus | -28, 60, 14 | 19 | 4.80 | <0.05 |

Abbreviations: R; Right, L; Left. * = whole brain, FWE corrected. No regions were significantly activated for the ‘Negative>Positive’ contrast term.

**Table S3.** Main effects of PPI for each seed region.

| **Seed Region** | **Contrast** | **Region** | **MNI Coordinates (X,Y,Z)** | ***k*-voxel** | ***z*-score** | ***p*-value*** |
|---|---|---|---|---|---|---|
| Left Anterior AI | Positive > neutral | | | | | |
| | | R Fusiform Gyrus | 40, -44, -20 | 2534 | 4.65 | <0.05 |
| | Negative > neutral | | | | | |
| | | R Fusiform Gyrus | 40, -58, -16 | 169 | 3.81 | <0.05 |
| Dorsal Anterior Cingulate Cortex | Positive > neutral | | | | | |
| | | R Fusiform Gyrus | 38, -72, -14 | 944 | 6.48 | <0.05 |
| | | L Inferior Occipital Lobe | -24, -98, -6 | 472 | 5.44 | <0.05 |

| | | | | | | |
|---|---|---|---|---|---|---|
| | | L Fusiform Gyrus | -36, -64, -14 | 452 | 5.14 | <0.05 |
| | Negative > neutral | | | | | |
| | | R Fusiform Gyrus | 40, -58, -16 | 1113 | 4.75 | <0.05 |

Abbreviations: R; Right, L; Left, *whole brain, FWE corrected.

**Table S4.** Main effects of the affect rating analysis (all whole brain, FWE corrected).

| Contrast | Region | MNI Coordinates (X,Y,Z) | *k*- voxels | *z*- score | *p*- value |
|---|---|---|---|---|---|
| Positive > Neutral | | | | | |
| | R Insula* | 30, 18, 18 | 228 | 4.77 | <0.05 |
| | Anterior Cingulate Cortex* | -2, 42, 12 | 2075 | 4,58 | <0.05 |
| Negative > Neutral | | | | | |
| | R Insula* | 28, 20, -16 | 113 | 6.00 | <0.05 |
| | L Anterior Cingulate* | -6, 36, 20 | | 5.71 | <0.05 |
| | R Superior Medial Frontal Gyrus | 8, 58, 26 | | 5.70 | <0.05 |
| | L Middle Temporal Gyrus* | -50, -32, -10 | 64 | 5.59 | <0.05 |
| | R Middle Temporal Gyrus | 60, -30, -6 | 76 | 5.41 | <0.05 |
| | L Insula* | -32, 16, -14 | 60 | 4.91 | <0.05 |
| | R Putamen | 26, 6, -8 | 55 | 4.86 | <0.05 |

Abbreviations: R; Right, L; Left, *; significant cluster identified in the conjunction analysis (p<0.001unc.) utilized for the exclusive masking of the Feedback Slide results.
